# Individualized design of thoracodorsal artery perforator chimeric flap for customized reconstruction of complex three-dimensional defects in the extremities

**DOI:** 10.1186/s13018-023-03852-z

**Published:** 2023-05-17

**Authors:** Liming Qing, Gaojie Luo, Xiaoxiao Li, Panfeng Wu, Juyu Tang

**Affiliations:** 1 Department of Microsurgery and Hand Surgery, Xiangya Hospital of Central South University, Changsha, 410008 China; 2 Department of Pathology, Changsha Medical University, Changsha, China

**Keywords:** Composite tissue defect, Chimeric flap, Thoracodorsal artery perforator flap

## Abstract

**Background:**

It was always challenging to accurately and effectively reconstruct the complicated defects with three-dimensional tissue deficits in the extremities. Muscle-chimeric perforator flap is an excellent choice for repairing those complicated wound. However, problems like donor-site morbidity and time-consuming intramuscular dissection still exist. This purpose of this study was to present a novel design of the thoracodorsal artery perforator (TDAP) chimeric flap for the customized reconstruction of complex three-dimensional tissue defects in the extremities.

**Methods:**

From January 2012 to June 2020, 17 patients with complex three-dimensional deficits in the extremities were retrospectively analyzed. All patients in this series underwent extremity reconstruction using latissimus dorsi (LD)-chimeric TDAP flap. Three different types of LD-chimeric TDAP flaps were performed.

**Results:**

A total of seventeen TDAP chimeric flaps were successfully harvested for the reconstruction of those complex three-dimensional defects in extremities. Among them, Design Type A flaps were used in 6 cases, Design Type B flaps were performed in 7 cases, and Design Type C flaps were used in the remaining 4 cases. The sizes of the skin paddles ranged from 6 cm × 3 cm to 24 cm × 11 cm. Meanwhile, the sizes of the muscle segments ranged from 3 cm × 4 cm to 33 cm × 4 cm. All the flaps survived. Nevertheless, one case required re-exploration owing to venous congestion. Moreover, the primary closure of the donor site was successfully achieved in all patients, and the mean follow-up time was 15.8 months. Most of the cases displayed satisfactory contour.

**Conclusion:**

The LD-chimeric TDAP flap is available for the reconstruction of complicated defects with three-dimensional tissue deficits in the extremities. It provided a flexible design for customized coverage of complex soft tissue defects with limited donor site morbidity.

## Introduction

High-energy trauma often leads to complicated wounds with three-dimensional deficits in the extremities involving superficial soft tissue defects and dead spaces of varying location, which are challenging to precisely and efficiently repair simultaneously [1–3]. The challenge of this type of reconstruction requires not only coverage of surface soft-tissue defects but also appropriate obliteration of dead space in a single procedure.

Numerous reconstructive strategies have been described for repairing complex three-dimensional soft tissue in the literature [4, 5]. Free latissimus dorsi muscle flaps have been widely accepted as a reliable option for reconstruct complicated defects with three-dimensionally inset multi-component tissue transfer. Nonetheless, reconstruction of this wound with a musculocutaneous flap always is more challenging for the reconstructive surgeon because of its restriction in the range of motion of muscle components. Moreover, those approaches are hindered by higher donor-site morbidity and poor contouring [6–9].

Recently, several studies have demonstrated that chimeric perforator flaps are one of the most valuable strategy for reconstructing complicated wounds with three-dimensional deficits, which can be attributed to their greater spatial freedom, more flexible design, the economy of donor incision, and superior aesthetic outcomes.[1, 10–13]. The latissimus dorsi (LD) muscle-chimeric thoracodorsal artery perforator (TDAP) flap has been introduced as a reliable strategy for repairing complicated wound [14]. However, harvesting chimeric perforator flaps with conventional design often lack the versatility to offer adequate tissue volume and allow precise tissue positioning to optimally cover the wound. Notably, when reconstructing complicated wounds with three-dimensional tissue deficits, customized chimeric perforator flap designs are necessitated for precisely coverage of superficial soft tissue defects and effective obliteration of dead space to facilitate wound healing. Moreover, the traditional designing of a LD muscle-chimeric TDAP flap typically requires time-consuming intramuscular dissection, leading to longer operative time and higher donor-site morbidity. To address this, three different types of LD muscle-chimeric TDAP flaps were designed to minimize donor-site morbidity and shorten the operative time. Thus, our report focused on the various designs for customized reconstruction of complex three-dimensional defects to minimize donor-site morbidity and shorten the operation time, which has rarely been addressed in previous studies. To the best of our knowledge, the use of individually designed chimeric perforator flaps to reconstruct complex three-dimensional soft tissue defects has not been studied in the context of free TDAP flaps.

The purpose of this study was to present our experience on a novel design of the TDAP chimeric flap and its various designs for the customized reconstruction of complex three-dimensional defects in the extremities.

## Patients and methods

Between January 2012 and June 2020, seventeen patients who suffered with complicated wound with three-dimensional tissue deficits were performed extremity reconstruction using a LD-muscle chimeric TDAP flap. Patient ages ranged from 2 to 39 years (mean age 20.24 years; 7 females, 10 males). Of these patients, five patients suffered from chronic osteomyelitis, two were injured by a crushing incident, three underwent tumor resection surgeries, and the remaining seven patients were injured in road traffic accidents. The complex soft-tissue defects were classified into three types according to the location of dead space. When the dead space was located at the center of the wound, it was classified as type I; when the dead space was located at the edge of the wound, it was classified as type II, while Type III was classified as a wound with extensive surface soft-tissue defect and dead space. Additionally, the type III category was further classified into two subtypes based on the location of dead space. When the dead space was located at the center of the wound, it was classified as type IIIA; when the dead space was at the edge of the wound, it was classified as type IIIB. Patient details are presented in Table 1. The study conformed to the ethical guidelines of the Hospital Ethical Committee of the Xiangya Hospital. The protocol was performed in accordance with the ethical standards of the Helsinki Declaration of 1975 and all subsequent revisions.

**Table 1:** Detailed information of the patients

Case	Age (y)/Sex	Cause	Type of defect	Type of design	Dimensions of skin paddle (cm)	Dimensions of muscle (cm)	Follow-up time (M)	Complications
1	25/F	Traffic accident	Type II	B	15 × 6	17 × 14	12	None
2	20/M	Traffic accident	Type I	A	19 × 7	15 × 5.5	8	None
3	29/M	Chronic Osteomyelitis	Type II	B	15 × 6	8 × 5	8	None
4	14/F	Tumor resections	Type II	B	15 × 7	8 × 3	24	None
5	20/M	Chronic Osteomyelitis	Type IIIB	C	10 × 6	8 × 4	18	None
6	19/F	Tumor resections	Type II	B	19 × 6	15 × 6	12	None
7	29/M	Crushing injures	Type IIIA	A	24 × 11	8 × 4	24	None
8	35/M	Chronic Osteomyelitis	Type I	A	19 × 6	33 × 4	12	Small-edge necrosis
9	39/M	traffic accident	Type II	B	15 × 9	12 × 6	15	None
10	72/M	Chronic Osteomyelitis	Type IIIA	A	21 × 7	32 × 3	24	None
11	19/F	Crushing injures	Type I	A	13 × 6	5 × 3	10	None
12	3/F	Traffic accident	Type I	A	13.5 × 5	11 × 4	24	Venous crisis
13	5/F	Chronic Osteomyelitis	Type IIIB	C	10 × 4.5	5 × 3	10	None
14	4/M	Traffic accident	Type IIIB	C	17 × 4	18 × 4	24	Small-edge necrosis
15	5/M	Traffic accident	Type II	B	10 × 6	5 × 2.5	24	None
16	2/F	Tumor resections	Type I	A	6 × 3	4 × 3	12	None
17	4/M	Traffic accident	Type IIIB	C	16 × 5	12 × 3.5	12	None

## Operative procedure

All patients were examined with an extremity computed tomography-assisted angiography (CTA) scan routinely to assess the vascular anatomy of the recipient site. The complicated wound was also assessed by our surgical team in order to perform a individually customized reconstructions. Following debridement, a paper template was created according to the dimensions of the soft tissue defect. A pinch test was performed to assess the maximum available skin size on the donor sites.

According to the characteristic of the complicated wound and the laxity of the skin over the lateral aspect of the dorsal thoracic, the volume of the muscle tissue segment was estimated based on the size of the dead space, which we aimed to eliminate at the recipient sites. This design protocol limited the width of each flap to less than the maximum available skin size, thereby achieving donor-site closure with low skin tension.

Based on the location of perforators mapped by hand Doppler probe preoperatively, the flap was outlined on the recipient site. Dissection was performed at the central portion of the superior border of the flap, followed by suprafascial dissection until the perforator was identified. Then, the perforator was traced back to the main trunk of the descending branch of the TDA, which was dissected according to pedicle length requirements. The thoracodorsal nerve was preserved as much as possible through thorough dissection, with the exception of the distal branch entering the harvested muscle segment.

**Fig. 1:**
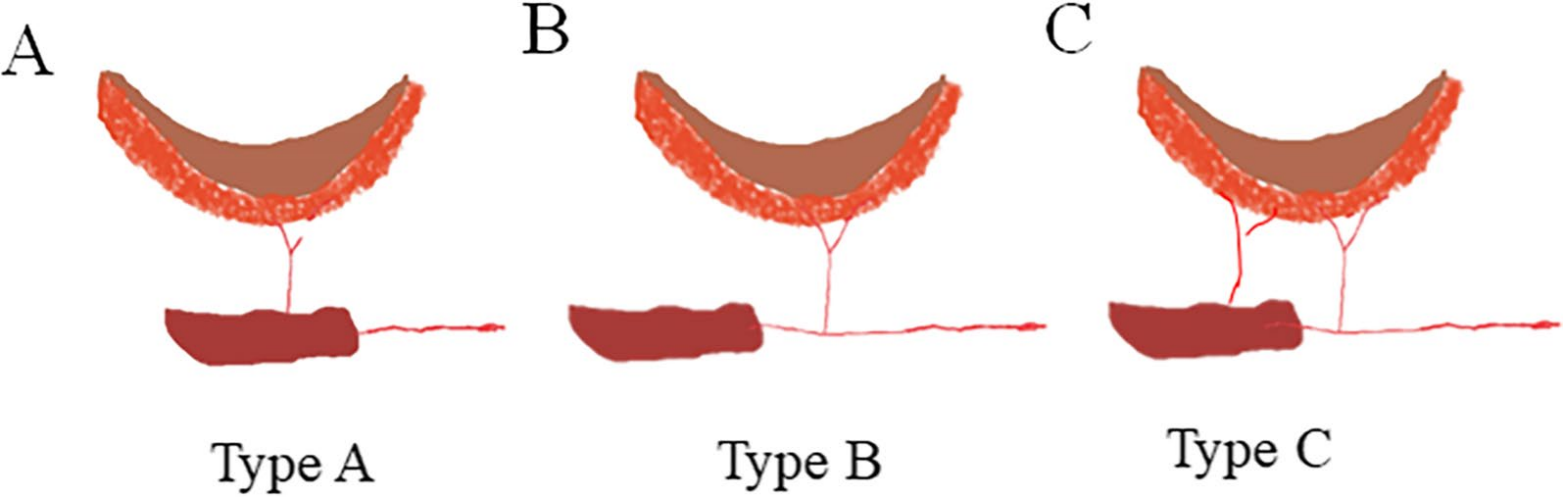
The flap could be harvested using one of the following surgical approaches (based on the desired design): **A** Design Type A: The vascular pedicle of the skin paddles through the muscle segment, and the muscle segment is nourished by the descending branch of the TDA. No time-consuming intramuscular dissection is required in this type of design. Therefore, this type design was the ability to shorten operative time for dissection of the flap and reduce donor-site morbidity. **B** Design Type B: the muscle segment is associated with one independent vessels branch from the source vessel that could be rotated up to 180°. It has longer vascular pedicle between the paddles and provides a greater degree of spatial freedom to avoid the pedicle kinking and twisting, but time-consuming intramuscular dissection is needed and higher donor site morbidity was unavoidable. **C** Design Type C: multiple perforator vessels were dissected to nourish the large skin paddle whose area exceeds the maximum size of one perforasome. The muscle segment is located on a side branch of one skin paddle or a separate branch from one of skin flap, the vascular pedicle of this skin paddle does not require intramuscular dissection. The other perforator vessels also have enough length of vascular pedicles to be freely three-dimensionally inset into the defect

The LD muscle-chimeric TDAP flaps were designed to include the independent skin paddle and muscle segments associated with the branch of the TDA. The flap was harvested using one of the following surgical approaches (based on the desired design) (Fig. 1):

Design Type A: the vascular pedicle of the skin paddled through the muscle tissue segment, and the muscle component was supplied by the main branch of the TDA. The skin paddles and muscle tissue segment were separated and only linked by the perforator vessels. No time-consuming intramuscular dissection of the perforator vessels was required for this design. Only a short vascular pedicle was kept between the skin paddles and muscle tissue segment was facilitated to achieve rotational or mobility of the skin paddles, which can precisely place these components to repair the complicated wound (Fig. 1A).

Design Type B: a LD muscle-chimeric TDAP flap with a Y-shaped pedicle configuration composed of a common pedicle and an independent pedicle for each component was harvested. When the muscle segment and skin paddle were supplied with an independent vessel branch from the TDA, a longer vascular pedicle and greater spatial freedom were expected. The vascular pedicle of each segment required time-consuming intramuscular dissection in this design (Fig. 1B).

Design Type C: mixed chimerism, multiple perforator vessels were dissected to supply blood to the large skin paddle whose area exceeded the maximum size of a perforasome. The muscle segment was located on a side branch of perforator vessels or a separate intramuscular branch from one perforator pedicle, this vascular pedicle did not require intramuscular dissection, whereas the remaining pedicles did. The vascular pedicles of the muscle paddles were sufficiently long to be freely three-dimensionally inserted into the dead space (Fig. 1C). After the flap was elevated, the muscle segment was inserted into the dead space, and the skin paddle was used to cover the surface soft tissue defects. After the flap was transferred to the recipient site, the wound of the donor site was directly closed.

## Results

For the repair of such three-dimensional defects in the extremities, a total of seventeen TDAP chimeric flaps were successfully harvested to. Flaps of Type A design were employed in six of them, Flaps of Type B design in seven, and Type C design in four of the cases. The size of skin paddles ranged from 6 cm × 3 cm to 24 cm × 11 cm. Meanwhile, the size of the muscle segment ranged from 3 cm × 4 cm to 33 cm × 4 cm. All the flaps survived. Only one flap required a second procedure due to venous congestion (Case 12). Two cases presented with minimal wound-edge necrosis and were conservatively managed (Cases 8, 14). The donor sites were directly closed without tension. The mean follow-up time was 15.8 months (ranged from 8 to 24 months). Most of cases presented with satisfactory contour, and excessive bulk was not observed.

### Case report

#### Case 4

**Fig. 2:**
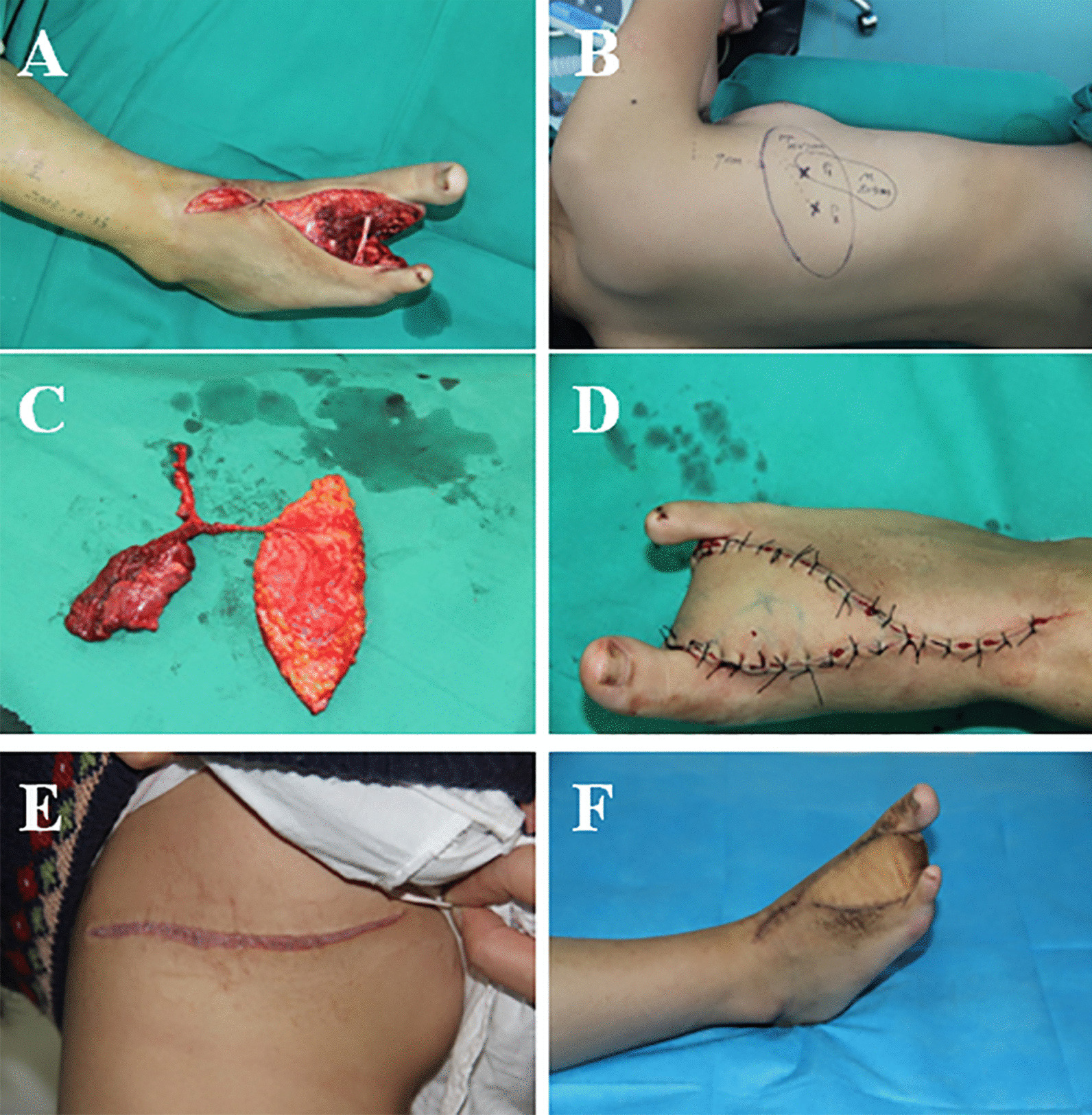
**A**, **B** A 14-year-old female with a large soft tissue defect, dead space, and exposure of extensor tendons and bone on the right foot. Radical debridement left Type II pattern defect. **C** Transversely oriented design of the LD muscle-chimeric TDAP flap. **D** Intraoperative view after harvest of the LD muscle-chimeric TDAP flap. **E**, **F** Postoperative view of the recipient site and donor site at 18-month follow-up

A 14-year-old female presented with a tumor on her right foot. A Type II pattern of the three-dimensional soft tissue defect was presented after radical debridement. A Type B pattern of muscle-chimeric TDAP flap was performed to repair the wound. A latissimus dorsi muscle paddle measuring 8 cm × 3 cm was harvested to eliminate the dead space. The donor and recipient areas recovered well after the operation (Fig. 2).

#### Case 10

**Fig. 3:**
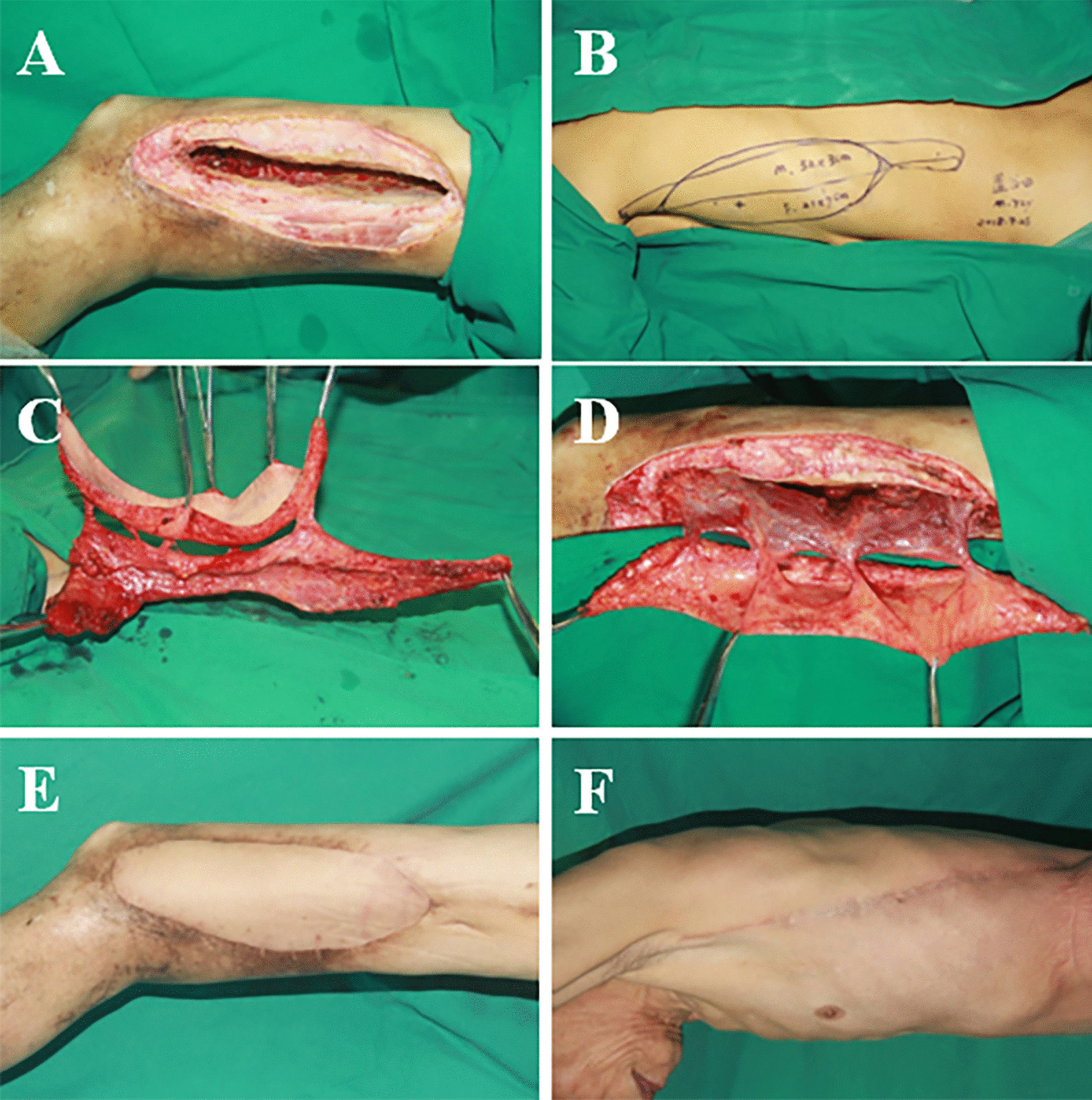
**A**, **B** A 72-year-old male suffered from chronic ulcer resulting in a large soft tissue defect, dead space, and exposure of tendons and bone on the left thigh. Radical debridement left Type I pattern defect. **C** Design of the LD muscle-chimeric TDAP flap. **D** Intraoperative view after harvest of the LD muscle-chimeric TDAP flap. **E**, **F** Postoperative view of the recipient site and donor site at 18-month follow-up

A 72-year-old male suffered from a chronic ulcer, which resulted in a large soft tissue defect and dead space on the right thigh. After radical debridement, A Type A pattern of muscle-chimeric TDAP flap was performed to reconstruct the complex three-dimensional soft tissue defect. The size of the skin paddles was 21 cm × 7 cm. A Latissimus dorsi muscle paddle measuring 32 cm × 3 cm was harvested to fill the dead space. No intramuscular dissection was required in this case. The postoperative course was uneventful, and the recipient site showed satisfactory contour (Fig. 3).

#### Case 17

**Fig. 4:**
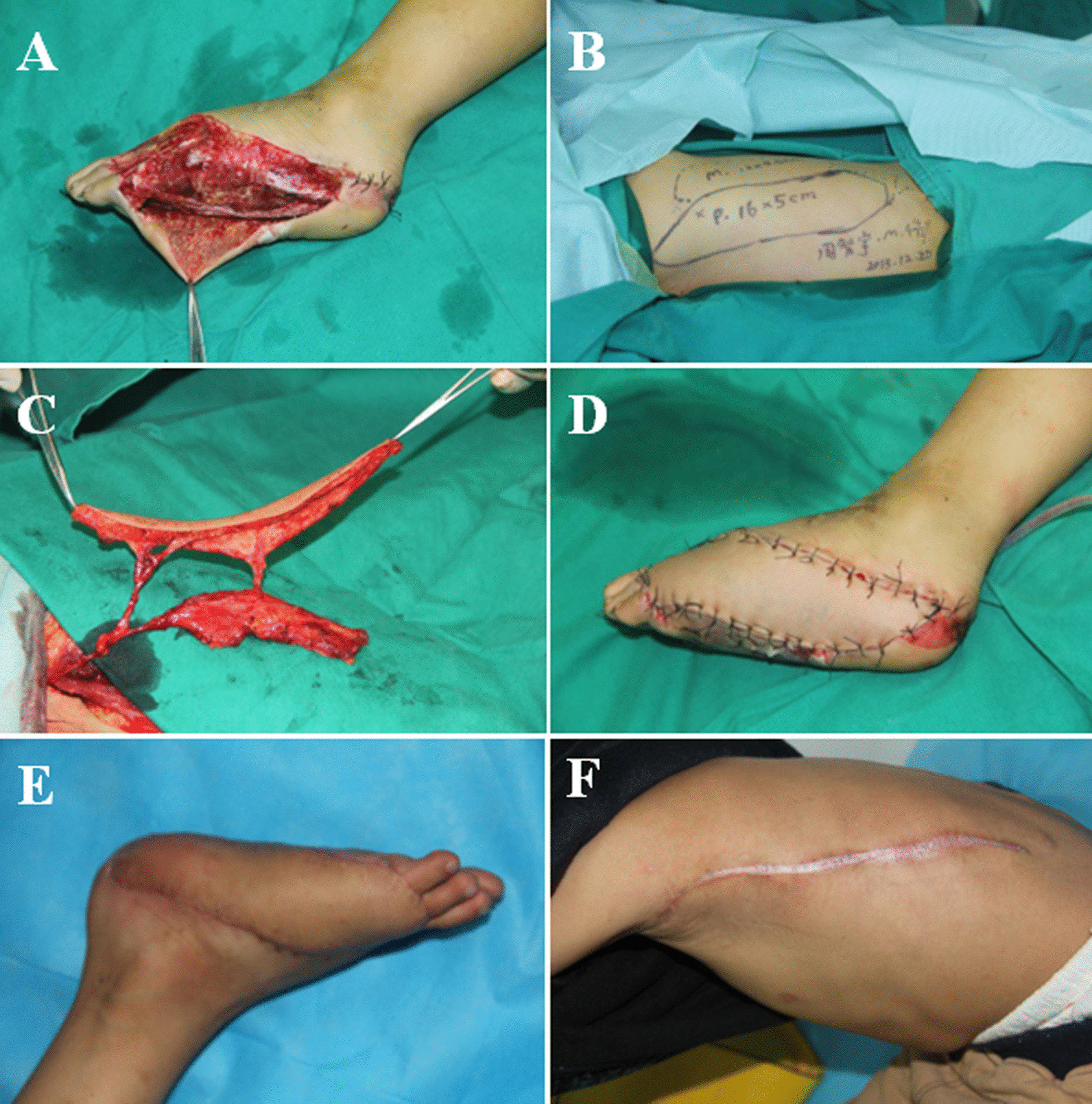
**A** A 4-year-old male with exposure of bone and dead space. **B**, **C** Design of the LD muscle-chimeric TDAP flap. **D** Intraoperative view after harvest of the LD muscle-chimeric TDAP flap. **E**, **F** Postoperative view of the recipient site and donor site

A 4-year-old child suffered from a traffic accident, which resulted in a large soft tissue defect on the left foot. To concurrently repair the large soft tissue defects and eliminate the dead space, a Type C pattern of the TDAP flap was designed. The size of the skin paddles was 16 cm × 5 cm. To obliterate the dead space, a muscle segment measuring 12 cm × 3.5 cm was harvested. The postoperative course was uneventful, and the recipient site showed satisfactory contour (Fig. 4).

## Discussion

Complicated wounds with three-dimensional deficits resulting from severe trauma, chronic infections, and tumor resections often present with superficial soft tissue defects and deep dead spaces [15–17]. One-stage reconstruction of the complex soft-tissue defects is essential to salvage an extremity, promote wound healing, and prevent postoperative complications. Elimination of dead space is essential, and failure to eliminate the dead space often results in necrosis of vascular flaps due to higher the risk of wound infection and hematoma. The mainstream approach uses muscle tissues to achieve the obliteration of dead space [12, 18]. The latissimus dorsi myocutaneous flap is currently the most popular traditional procedure to repair a complicated wound owing to benefits such as a concealed donor site, availability of multiple blood vessels, a large available area for incision, rich blood supply, and potent anti-infection ability [12, 19, 20]. However, this procedure failed to separate the muscle and the skin, which results in the limitation of freedom of movement; actually, it could not achieve accurate obliteration of the dead space. Besides, excessive muscle tissue incision also causes severe injury to the donor site and an unsightly appearance. The combined transplantation of free muscle tissue and skin flaps is another potential strategy for repairing complicated wounds with three-dimensional deficits [16]. However, this method requires anastomosis of two sets of blood vessels, and the muscle segment and skin flap need to be harvested from two donor sites, which prolongs the operation time, damages the donor site as well as elevates the risk of surgery.

In view of the shortcomings of the above-mentioned traditional methods, the concept of chimeric perforator flap has been proposed in recent years as a possible approach to reconstruct complicated three-dimensional tissue deficits [21–23]. The nomenclature was unclear until Huang [24] and Hallock [25] defined this flap as a chimeric perforator flap in 2003. Following this, Hallock further systematically introduced the chimeric concept of perforator flaps based on different blocks of tissue were nourished by different branches of vessels with a common pedicle [26]. Notably, the muscle-chimeric perforator flap offers numerous advantages for repairing complicated tissue defects: ① Only a set of vessels pedicle requires anastomosis to achieve not only successful coverage of surface soft tissue defects with skin paddle but also effective elimination of dead space with muscle segments; ② the muscle segment can be accurately selected according to the volume of the dead space while minimizing donor site morbidity; ③The muscle segment and the skin paddle can be independently and easily inserted with more spatial freedom, which facilitate to avoid the folding and twisting of the pedicle and the tissue, thereby resulting in an enhanced an aesthetic appearance; ④ Muscle tissues are known to improve local blood supply, increase oxygen tension and antibiotic delivery to wounds, leading to faster wound healing. Muscle tissues easily adapt to the shape of the defect and are thus optimal for the elimination of dead space in the wound.

Although several advantages of the muscle-chimeric perforator flap have been documented in the previous literature, the conventional design of chimeric flaps has focused on the simultaneous harvest of multiple types of tissues without paying particular attention to the pedicle length of each component and has mainly been applied for the coverage of large and extensive defects. In addition, the traditional design of chimeric flaps lacks the versatility to offer adequate tissue volume and allow precise tissue positioning to optimally cover the wound. To efficiently cover the surface defect and at the same time obliterate the underlying dead space for complex three-dimensional soft tissue defects, a variant of the design of the muscle-chimeric perforator flap is warranted. Herein, we presented our insights into utilizing individual designs of chimeric TDAP flaps to reconstruct complicated three-dimensional soft tissue defects. We also introduced a novel classification system of chimeric perforator flaps for reconstructive surgeons and trainees to gain a better understanding of chimeric flaps design and allow safe, effective, and aesthetically superior reconstruction of complex three-dimensional defects.

Various donor sites, including the thoracodorsal artery (TDA), subscapular vessel [27, 28], lateral circumflex femoral vessel (LCFV) [29], and deep inferior epigastric vessel (DIEV) [30] systems, have been described in the literature for harvesting chimeric perforator flaps. Among them, the gold standard for the reconstruction of complicated soft tissue defects in adults remains the vastus lateralis muscle-chimeric anterolateral thigh perforator (ALTP) free flap [29]. However, the ALTP flap is well known for variations in its vascular pedicle, and failure to understand its variability can lead to vascular flap embarrassment and tissue loss [31, 32]. Recently emerge researches have demonstrated that a suitable cutaneous perforator vessels for free ALTP flap transfer in the thigh was absent in 5–6% of patients [33, 34]. Muscle-chimeric TDAP flap possesses the inherent advantages of latissimus dorsi myocutaneous flaps, such as a concealed donor site, large available area for incision, strong anti-infection ability, and rich blood supply, and thus is a useful alternative for reconstructing complex three-dimensional soft tissue defects. However, the majority of studies have reported that the muscle component was predominantly used to increase the dimensional of flap for covering huge soft tissue defect, and providing a well-vascularized bed for skin grafts when the wound could not be covered with the skin perforator flap alone. Studies regarding the use of this flap for reconstructing complex three-dimensional extremity defects are limited. Recently, Lee KT et al. [12] reported the use of free latissimus dorsi muscle-chimeric TDAP flaps for the reconstruction of complex soft tissue defects and emphasized on obtaining a Y-shaped pedicle configuration with adequate lengths for the skin paddle and the muscle segment; as expected, meticulous intramuscular dissection was needed for both tissue segments. Therefore, a time-consuming intramuscular dissection was unavoidable for the acquisition of the pedicle, which led to higher donor site morbidity. In this study, three types of flap designs were developed for the customized reconstruction of complex three-dimensional tissue defects. Our report focused on the novel design of chimeric TDAP flap and its various designs for the individualized reconstruction of three-dimensional defects to minimize the donor site morbidity and shorten the operation time. To the best of our knowledge, these are the largest series to date reporting microvascular reconstruction of complicated three-dimensional soft-tissue defects in the extremities using customized designed chimeric TDAP flaps.

**Fig. 5:**
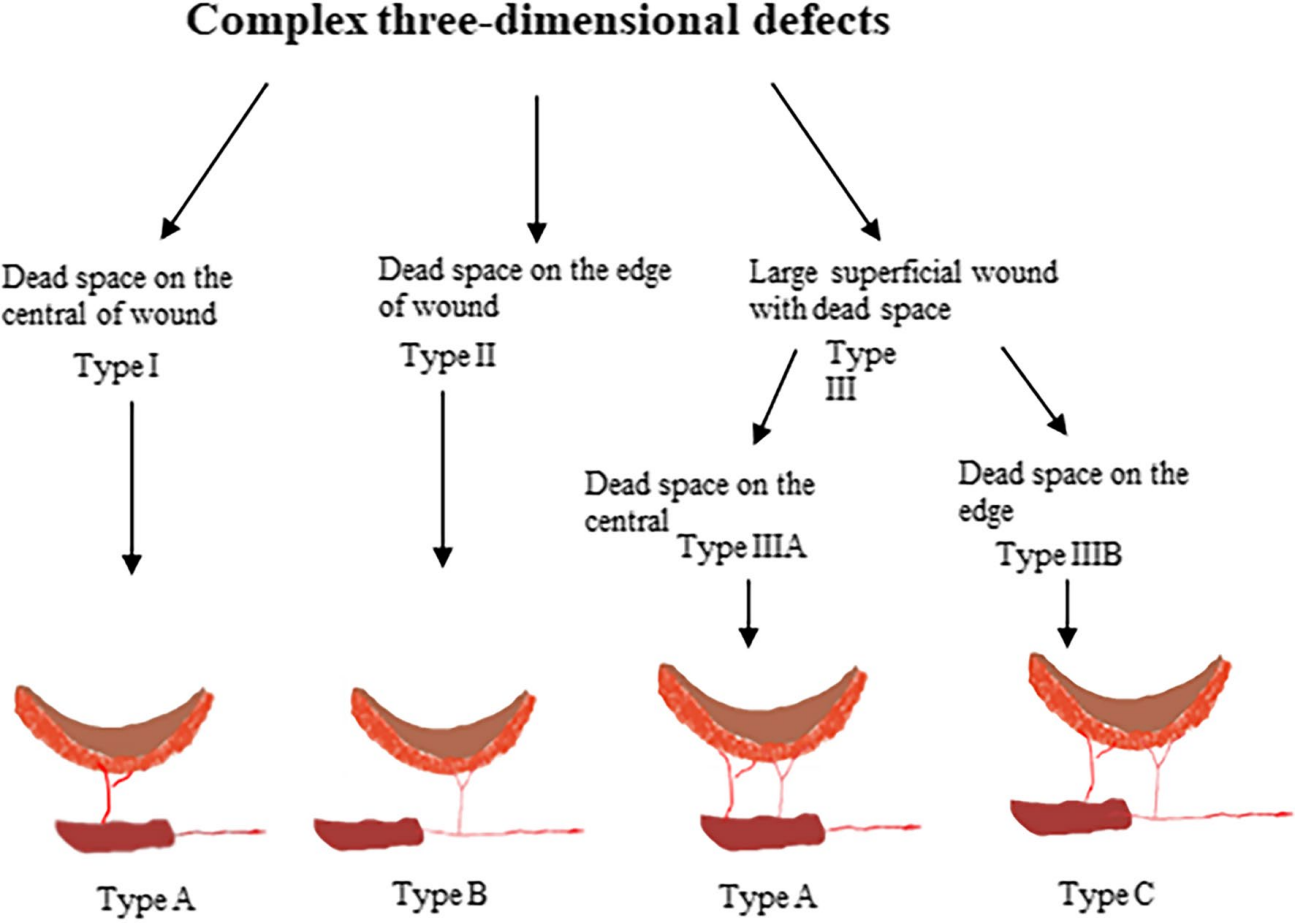
Diagram illustrated a working algorithm for the reconstruction of complex three-dimensional defects by using the TDAP chimeric flap

Based on our previous clinical work experience, a working algorithm can be introduced for the reconstruction of complex three-dimensional defects using the TDAP chimeric flap (Fig. 5). The type of surgical procedure is determined by several factors, including the wound size, location of dead space, and the degree of morbidity at the donor site. When the dead space located on the center of wound, only a short vascular pedicle which linked the muscle component and the skin paddle was required to achieve freedom mobilization or rotational of the skin paddles. It was facilitated to precisely insert the muscle into dead space and place the skin paddles to cover the superficial soft tissue defect. Thus, the primary objective of reconstruction is to reduce intramuscular dissection of the perforator to reduce donor site morbidity and shorten the operation time. In this context, type A design is the optimal choice. A short operation time, as well as limited site morbidity, are the expected benefits. In contrast, when the dead space is located on the edge of the wound, a longer vascular pedicle is required for the muscle component and the skin paddles to effectively reconstruct the three-dimensional soft tissue defect. Moreover, to reduce pedicle tension and twisting, the length of the vascular pedicle between components should also be completely taken into consideration. In this situation, a type B design might be more appropriate which presented with a longer vascular pedicle between components and more freedom to avoid pedicle kinking and twisting. It is worthwhile to point out that even though the type B design was still unable to avoid high morbidity of the donor site and time-consuming intramuscular dissection. Eventually, ischemic necrosis of the distal flap portion remains a commonly encountered complication in clinical practice for the reconstruction of larger complicated wound, and the balance between donor-site morbidity and optimal reconstruction of recipient sites needs to be carefully assessed. Type C may potentially be more suitable in this situation. This type design presented with multiple perforator vessels to supply blood to the large skin paddle which have been proven to enhance the flap's viability in clinical practice. However, considering that the muscle segment is located on a side branch whereby no tedious intramuscular dissection is required, whilst other perforator vessels still need to be meticulously dissected to harvest sufficiently long vascular pedicles to be freely three-dimensional insets.

If the three types of flap design were suitable for the wound, the primary aim of the approach is to reduce the donor site's morbidity and scar-related disorders. We prioritized choosing a transverse-designed skin paddle flap on this donor site for the moderate-sized soft tissue defects. Comparing transverse-oriented flaps to non-transverse-oriented flaps, several studies have found that transverse-oriented flaps on the backside significantly reduce the risk of developing scar-related issues at the donor site [35]. Our previous study also demonstrated that the transverse design of the scapular artery perforator flap presented a more aesthetic appearance. Moreover, the transverse design causes less lateral movement of breast mounds and nipples during donor-site closure. Considering the donor site's cosmetic appearance improvement, the recent practice showed that using a transverse designed flap was more effective than using a non-transverse one. Also, for female patients, the scar can be readily concealed with a brassiere.

The limitations of this study include the small number of cases as well as its retrospective character. And other limitations include the absence of a direct comparison group and a standardized assessment tool for the objective assessment of the result following reconstructive surgery. Thus, further studies, such as prospective case–control or randomized studies, are needed in future study.

## Conclusions

To summarize, the TDA chimeric perforator flap is a reliable option for the reconstruction of complex three-dimensional defects of the extremities. This flexible approach provides various designs for customized coverage of complex three-dimensional defects with limited donor site morbidity. Moreover, the flexible shape of this flap permits alteration to match the shape of the recipient site. In addition, we introduced a novel classification system of chimeric perforator flaps for reconstructive surgeons and trainees to gain a better understanding of chimeric flaps design and allow safe, effective, and aesthetically superior reconstruction of complex three-dimensional defects.

## Data Availability

Data sets or analyses used in the current study are available from the corresponding author upon reasonable request.
